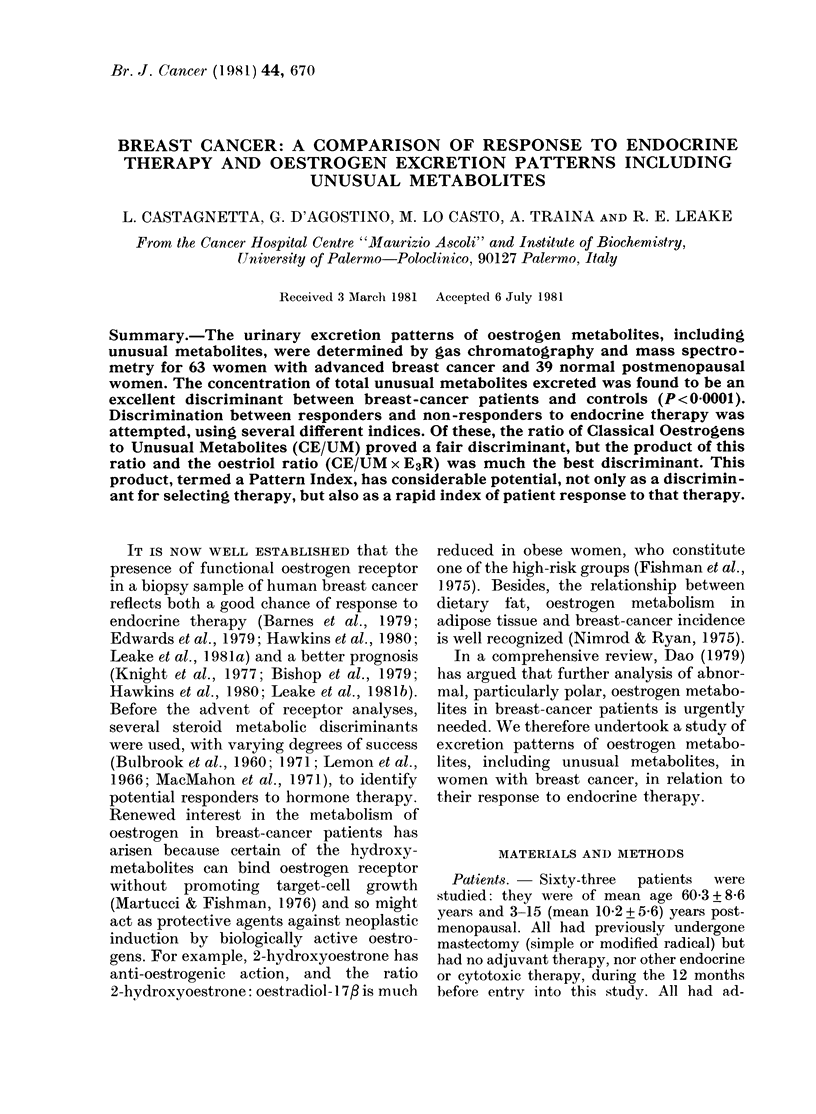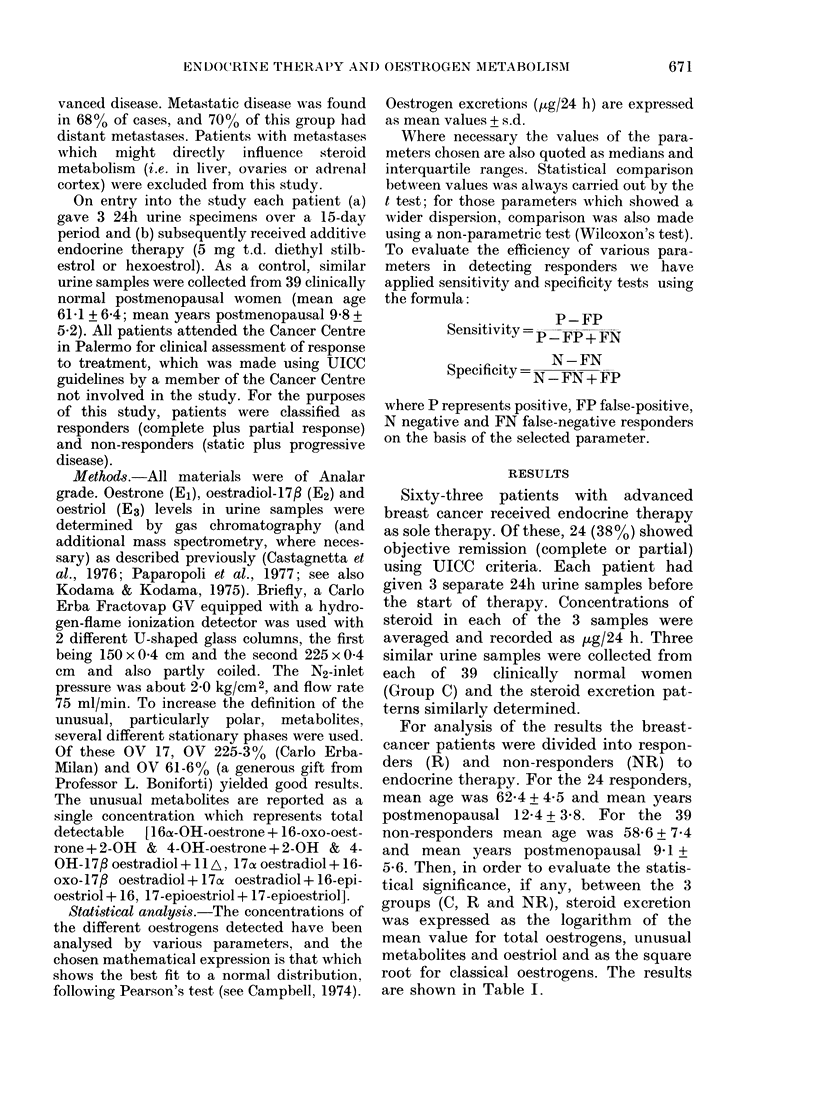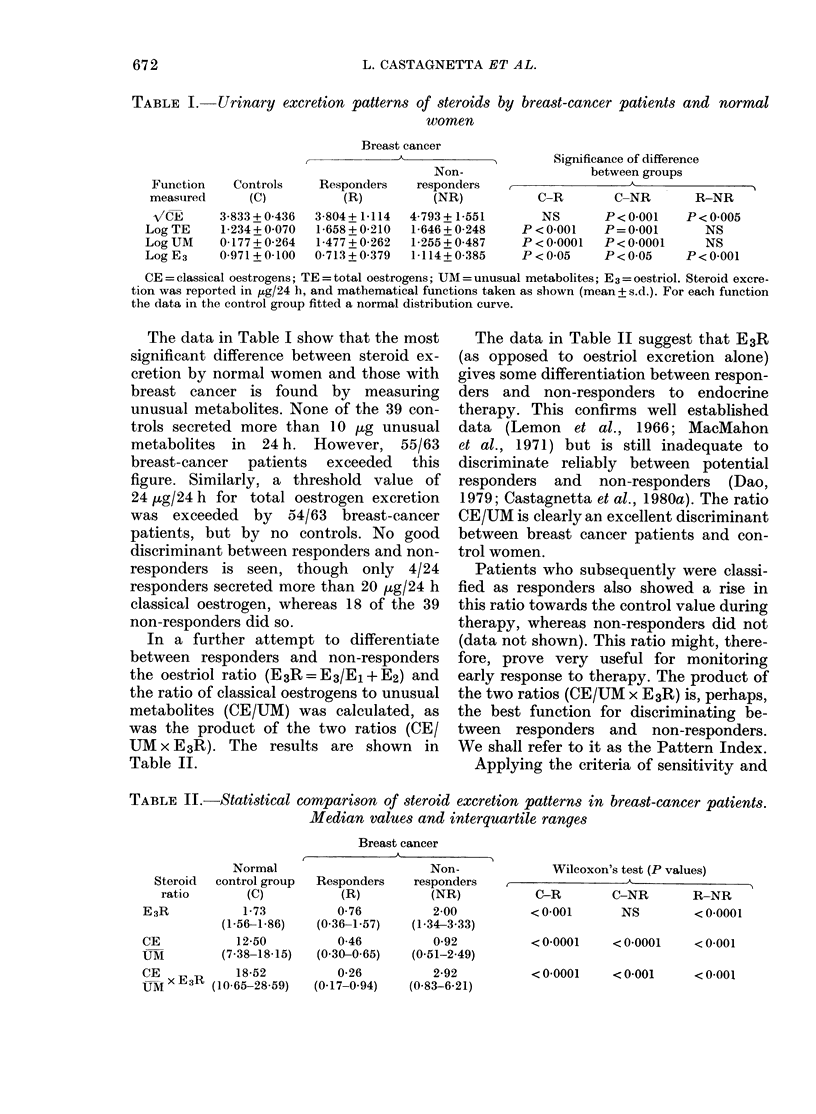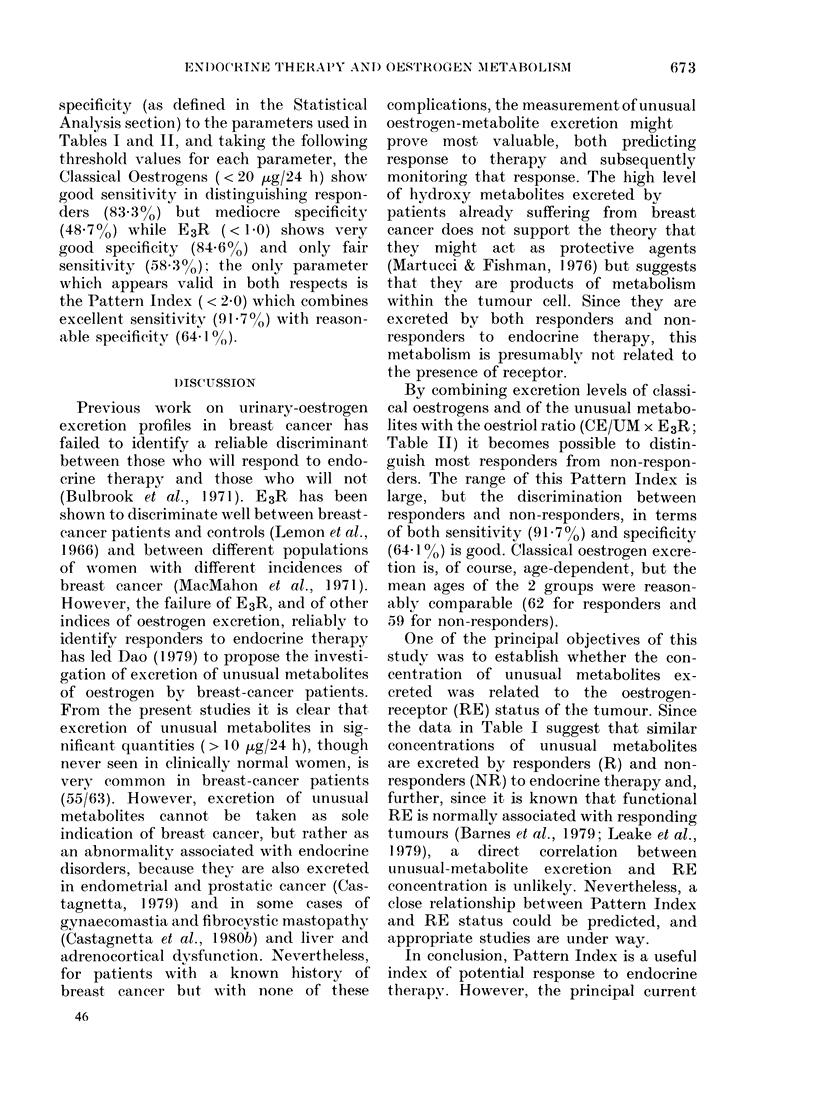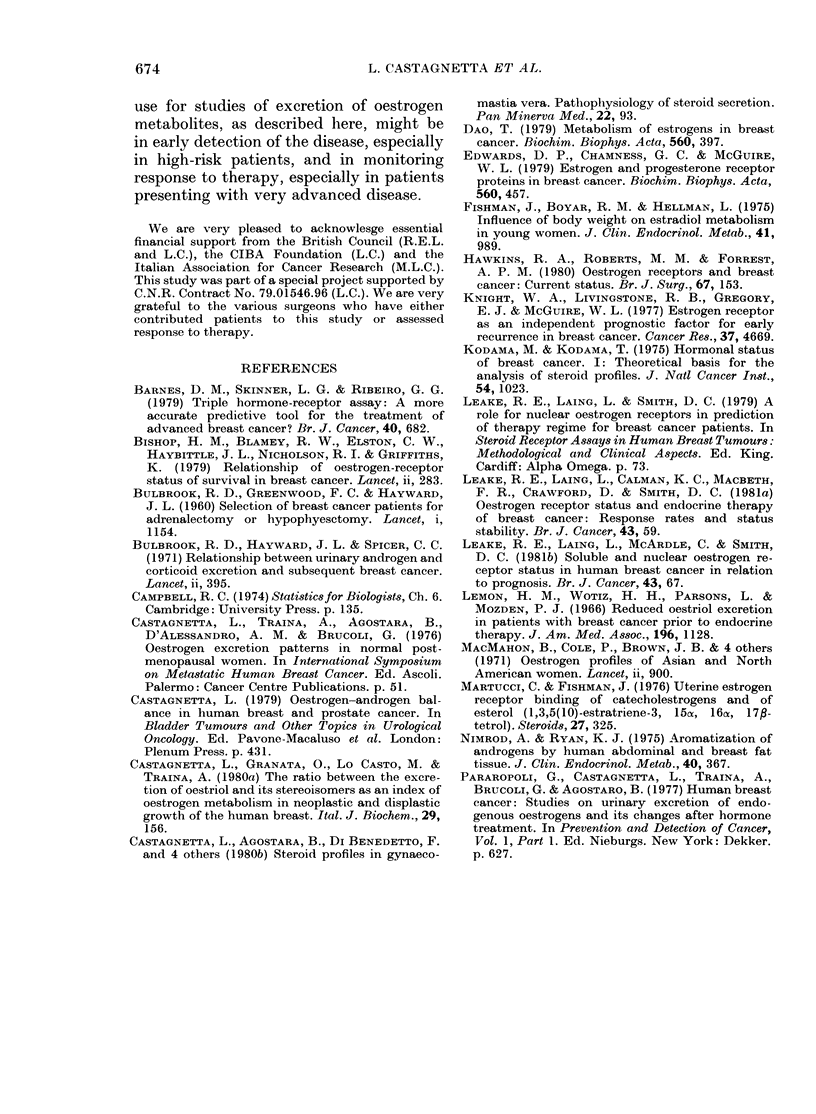# Breast cancer: a comparison of response to endocrine therapy and oestrogen excretion patterns including unusual metabolites.

**DOI:** 10.1038/bjc.1981.252

**Published:** 1981-11

**Authors:** L. Castagnetta, C. D'Agostino, M. Lo Casto, A. Traina, R. E. Leake

## Abstract

The urinary excretion patterns of oestrogen metabolites, including unusual metabolites, were determined by gas chromatography and mass spectrometry for 63 women with advanced breast cancer and 39 normal postmenopausal women. The concentration of total unusual metabolites excreted was found to be an excellent discriminant between breast-cancer patients and controls (P less than 0.0001). Discrimination between responders and non-responders to endocrine therapy was attempted, using several different indices. Of these, the ratio of Classical Oestrogens to Unusual Metabolites (CE/UM) proved a fair discriminant, but the product of this ratio and the oestriol ratio (CE/UM x E3R) was much the best discriminant. This product, termed a Pattern Index, has considerable potential, not only as a discriminant for selecting therapy, but also as a rapid index of patient response to that therapy.


*Br. J. Cancer* (1981) **44**, 670

### BREAST CANCER: A COMPARISON OF RESPONSE TO ENDOCRINE THERAPY AND OESTROGEN EXCRETION PATTERNS INCLUDING UNUSUAL METABOLITES

L. CASTAGNETTA, G. D'AGOSTINO, M. LO CASTO, A. TRAINA AND R. E. LEAKE

*From the Cancer Hospital Centre "Maurizio Ascoli" and Institute of Biochemistry, University of Palermo—Poloclinico,* 90127 *Palermo, Italy*

Received 3 March 1981 Accepted 6 July 1981

**Summary.—The urinary excretion patterns of oestrogen metabolites, including unusual metabolites, were determined by gas chromatography and mass spectrometry for 63 women with advanced breast cancer and 39 normal postmenopausal women. The concentration of total unusual metabolites excreted was found to be an excellent discriminant between breast-cancer patients and controls ($P<0{\cdot}0001$). Discrimination between responders and non-responders to endocrine therapy was attempted, using several different indices. Of these, the ratio of Classical Oestrogens to Unusual Metabolites (CE/UM) proved a fair discriminant, but the product of this ratio and the oestriol ratio (CE/UM × $E_3R$) was much the best discriminant. This product, termed a Pattern Index, has considerable potential, not only as a discriminant for selecting therapy, but also as a rapid index of patient response to that therapy.**

IT IS NOW WELL ESTABLISHED that the presence of functional oestrogen receptor in a biopsy sample of human breast cancer reflects both a good chance of response to endocrine therapy (Barnes *et al.*, 1979; Edwards *et al.*, 1979; Hawkins *et al.*, 1980; Leake *et al.*, 1981*a*) and a better prognosis (Knight *et al.*, 1977; Bishop *et al.*, 1979; Hawkins *et al.*, 1980; Leake *et al.*, 1981*b*). Before the advent of receptor analyses, several steroid metabolic discriminants were used, with varying degrees of success (Bulbrook *et al.*, 1960; 1971; Lemon *et al.*, 1966; MacMahon *et al.*, 1971), to identify potential responders to hormone therapy. Renewed interest in the metabolism of oestrogen in breast-cancer patients has arisen because certain of the hydroxy-metabolites can bind oestrogen receptor without promoting target-cell growth (Martucci & Fishman, 1976) and so might act as protective agents against neoplastic induction by biologically active oestrogens. For example, 2-hydroxyoestrone has anti-oestrogenic action, and the ratio 2-hydroxyoestrone: oestradiol-17$\beta$ is much reduced in obese women, who constitute one of the high-risk groups (Fishman *et al.*, 1975). Besides, the relationship between dietary fat, oestrogen metabolism in adipose tissue and breast-cancer incidence is well recognized (Nimrod & Ryan, 1975).

In a comprehensive review, Dao (1979) has argued that further analysis of abnormal, particularly polar, oestrogen metabolites in breast-cancer patients is urgently needed. We therefore undertook a study of excretion patterns of oestrogen metabolites, including unusual metabolites, in women with breast cancer, in relation to their response to endocrine therapy.

### MATERIALS AND METHODS

*Patients.* — Sixty-three patients were studied: they were of mean age $60{\cdot}3 \pm 8{\cdot}6$ years and 3–15 (mean $10{\cdot}2 \pm 5{\cdot}6$) years post-menopausal. All had previously undergone mastectomy (simple or modified radical) but had no adjuvant therapy, nor other endocrine or cytotoxic therapy, during the 12 months before entry into this study. All had advanced disease. Metastatic disease was found in 68% of cases, and 70% of this group had distant metastases. Patients with metastases which might directly influence steroid metabolism (*i.e.* in liver, ovaries or adrenal cortex) were excluded from this study.

On entry into the study each patient (a) gave 3 24h urine specimens over a 15-day period and (b) subsequently received additive endocrine therapy (5 mg t.d. diethyl stilbestrol or hexoestrol). As a control, similar urine samples were collected from 39 clinically normal postmenopausal women (mean age 61·1 ± 6·4; mean years postmenopausal 9·8 ± 5·2). All patients attended the Cancer Centre in Palermo for clinical assessment of response to treatment, which was made using UICC guidelines by a member of the Cancer Centre not involved in the study. For the purposes of this study, patients were classified as responders (complete plus partial response) and non-responders (static plus progressive disease).

*Methods.*—All materials were of Analar grade. Oestrone ($E_1$), oestradiol-17$\beta$ ($E_2$) and oestriol ($E_3$) levels in urine samples were determined by gas chromatography (and additional mass spectrometry, where necessary) as described previously (Castagnetta *et al.*, 1976; Paparopoli *et al.*, 1977; see also Kodama & Kodama, 1975). Briefly, a Carlo Erba Fractovap GV equipped with a hydrogen-flame ionization detector was used with 2 different U-shaped glass columns, the first being 150 × 0·4 cm and the second 225 × 0·4 cm and also partly coiled. The $N_2$-inlet pressure was about 2·0 kg/cm$^2$, and flow rate 75 ml/min. To increase the definition of the unusual, particularly polar, metabolites, several different stationary phases were used. Of these OV 17, OV 225-3% (Carlo Erba-Milan) and OV 61-6% (a generous gift from Professor L. Boniforti) yielded good results. The unusual metabolites are reported as a single concentration which represents total detectable [16$\alpha$-OH-oestrone + 16-oxo-oestrone + 2-OH & 4-OH-oestrone + 2-OH & 4-OH-17$\beta$ oestradiol + 11$\triangle$, 17$\alpha$ oestradiol + 16-oxo-17$\beta$ oestradiol + 17$\alpha$ oestradiol + 16-epi-oestriol + 16, 17-epioestriol + 17-epioestriol].

*Statistical analysis.*—The concentrations of the different oestrogens detected have been analysed by various parameters, and the chosen mathematical expression is that which shows the best fit to a normal distribution, following Pearson's test (see Campbell, 1974). Oestrogen excretions ($\mu$g/24 h) are expressed as mean values ± s.d.

Where necessary the values of the parameters chosen are also quoted as medians and interquartile ranges. Statistical comparison between values was always carried out by the *t* test; for those parameters which showed a wider dispersion, comparison was also made using a non-parametric test (Wilcoxon's test). To evaluate the efficiency of various parameters in detecting responders we have applied sensitivity and specificity tests using the formula:

$$\text{Sensitivity} = \frac{P - FP}{P - FP + FN}$$

$$\text{Specificity} = \frac{N - FN}{N - FN + FP}$$

where P represents positive, FP false-positive, N negative and FN false-negative responders on the basis of the selected parameter.

### RESULTS

Sixty-three patients with advanced breast cancer received endocrine therapy as sole therapy. Of these, 24 (38%) showed objective remission (complete or partial) using UICC criteria. Each patient had given 3 separate 24h urine samples before the start of therapy. Concentrations of steroid in each of the 3 samples were averaged and recorded as $\mu$g/24 h. Three similar urine samples were collected from each of 39 clinically normal women (Group C) and the steroid excretion patterns similarly determined.

For analysis of the results the breast-cancer patients were divided into responders (R) and non-responders (NR) to endocrine therapy. For the 24 responders, mean age was 62·4 ± 4·5 and mean years postmenopausal 12·4 ± 3·8. For the 39 non-responders mean age was 58·6 ± 7·4 and mean years postmenopausal 9·1 ± 5·6. Then, in order to evaluate the statistical significance, if any, between the 3 groups (C, R and NR), steroid excretion was expressed as the logarithm of the mean value for total oestrogens, unusual metabolites and oestriol and as the square root for classical oestrogens. The results are shown in Table I.

TABLE I.—*Urinary excretion patterns of steroids by breast-cancer patients and normal women*

| Function measured | Controls (C) | Breast cancer: Responders (R) | Breast cancer: Non-responders (NR) | Significance of difference between groups: C–R | C–NR | R–NR |
|---|---|---|---|---|---|---|
| $\sqrt{CE}$ | $3 \cdot 833 \pm 0 \cdot 436$ | $3 \cdot 804 \pm 1 \cdot 114$ | $4 \cdot 793 \pm 1 \cdot 551$ | NS | $P < 0 \cdot 001$ | $P < 0 \cdot 005$ |
| Log TE | $1 \cdot 234 \pm 0 \cdot 070$ | $1 \cdot 658 \pm 0 \cdot 210$ | $1 \cdot 646 \pm 0 \cdot 248$ | $P < 0 \cdot 001$ | $P = 0 \cdot 001$ | NS |
| Log UM | $0 \cdot 177 \pm 0 \cdot 264$ | $1 \cdot 477 \pm 0 \cdot 262$ | $1 \cdot 255 \pm 0 \cdot 487$ | $P < 0 \cdot 0001$ | $P < 0 \cdot 0001$ | NS |
| Log $E_3$ | $0 \cdot 971 \pm 0 \cdot 100$ | $0 \cdot 713 \pm 0 \cdot 379$ | $1 \cdot 114 \pm 0 \cdot 385$ | $P < 0 \cdot 05$ | $P < 0 \cdot 05$ | $P < 0 \cdot 001$ |

CE = classical oestrogens; TE = total oestrogens; UM = unusual metabolites; $E_3$ = oestriol. Steroid excretion was reported in μg/24 h, and mathematical functions taken as shown (mean ± s.d.). For each function the data in the control group fitted a normal distribution curve.

The data in Table I show that the most significant difference between steroid excretion by normal women and those with breast cancer is found by measuring unusual metabolites. None of the 39 controls secreted more than 10 μg unusual metabolites in 24 h. However, 55/63 breast-cancer patients exceeded this figure. Similarly, a threshold value of 24 μg/24 h for total oestrogen excretion was exceeded by 54/63 breast-cancer patients, but by no controls. No good discriminant between responders and non-responders is seen, though only 4/24 responders secreted more than 20 μg/24 h classical oestrogen, whereas 18 of the 39 non-responders did so.

In a further attempt to differentiate between responders and non-responders the oestriol ratio ($E_3R = E_3/E_1 + E_2$) and the ratio of classical oestrogens to unusual metabolites (CE/UM) was calculated, as was the product of the two ratios (CE/UM × $E_3$R). The results are shown in Table II.

The data in Table II suggest that $E_3$R (as opposed to oestriol excretion alone) gives some differentiation between responders and non-responders to endocrine therapy. This confirms well established data (Lemon *et al.*, 1966; MacMahon *et al.*, 1971) but is still inadequate to discriminate reliably between potential responders and non-responders (Dao, 1979; Castagnetta *et al.*, 1980*a*). The ratio CE/UM is clearly an excellent discriminant between breast cancer patients and control women.

Patients who subsequently were classified as responders also showed a rise in this ratio towards the control value during therapy, whereas non-responders did not (data not shown). This ratio might, therefore, prove very useful for monitoring early response to therapy. The product of the two ratios (CE/UM × $E_3$R) is, perhaps, the best function for discriminating between responders and non-responders. We shall refer to it as the Pattern Index.

Applying the criteria of sensitivity and

TABLE II.—*Statistical comparison of steroid excretion patterns in breast-cancer patients. Median values and interquartile ranges*

| Steroid ratio | Normal control group (C) | Breast cancer: Responders (R) | Breast cancer: Non-responders (NR) | Wilcoxon's test (*P* values): C–R | C–NR | R–NR |
|---|---|---|---|---|---|---|
| $E_3$R | 1·73 (1·56–1·86) | 0·76 (0·36–1·57) | 2·00 (1·34–3·33) | <0·001 | NS | <0·0001 |
| $\frac{CE}{UM}$ | 12·50 (7·38–18·15) | 0·46 (0·30–0·65) | 0·92 (0·51–2·49) | <0·0001 | <0·0001 | <0·001 |
| $\frac{CE}{UM} \times E_3R$ | 18·52 (10·65–28·59) | 0·26 (0·17–0·94) | 2·92 (0·83–6·21) | <0·0001 | <0·001 | <0·001 |

specificity (as defined in the Statistical Analysis section) to the parameters used in Tables I and II, and taking the following threshold values for each parameter, the Classical Oestrogens ($<20$ $\mu$g/24 h) show good sensitivity in distinguishing responders (83·3%) but mediocre specificity (48·7%) while $E_3R$ ($<1·0$) shows very good specificity (84·6%) and only fair sensitivity (58·3%); the only parameter which appears valid in both respects is the Pattern Index ($<2·0$) which combines excellent sensitivity (91·7%) with reasonable specificity (64·1%).

### DISCUSSION

Previous work on urinary-oestrogen excretion profiles in breast cancer has failed to identify a reliable discriminant between those who will respond to endocrine therapy and those who will not (Bulbrook *et al.*, 1971). $E_3R$ has been shown to discriminate well between breast-cancer patients and controls (Lemon *et al.*, 1966) and between different populations of women with different incidences of breast cancer (MacMahon *et al.*, 1971). However, the failure of $E_3R$, and of other indices of oestrogen excretion, reliably to identify responders to endocrine therapy has led Dao (1979) to propose the investigation of excretion of unusual metabolites of oestrogen by breast-cancer patients. From the present studies it is clear that excretion of unusual metabolites in significant quantities ($>10$ $\mu$g/24 h), though never seen in clinically normal women, is very common in breast-cancer patients (55/63). However, excretion of unusual metabolites cannot be taken as sole indication of breast cancer, but rather as an abnormality associated with endocrine disorders, because they are also excreted in endometrial and prostatic cancer (Castagnetta, 1979) and in some cases of gynaecomastia and fibrocystic mastopathy (Castagnetta *et al.*, 1980*b*) and liver and adrenocortical dysfunction. Nevertheless, for patients with a known history of breast cancer but with none of these complications, the measurement of unusual oestrogen-metabolite excretion might prove most valuable, both predicting response to therapy and subsequently monitoring that response. The high level of hydroxy metabolites excreted by patients already suffering from breast cancer does not support the theory that they might act as protective agents (Martucci & Fishman, 1976) but suggests that they are products of metabolism within the tumour cell. Since they are excreted by both responders and non-responders to endocrine therapy, this metabolism is presumably not related to the presence of receptor.

By combining excretion levels of classical oestrogens and of the unusual metabolites with the oestriol ratio ($CE/UM \times E_3R$; Table II) it becomes possible to distinguish most responders from non-responders. The range of this Pattern Index is large, but the discrimination between responders and non-responders, in terms of both sensitivity (91·7%) and specificity (64·1%) is good. Classical oestrogen excretion is, of course, age-dependent, but the mean ages of the 2 groups were reasonably comparable (62 for responders and 59 for non-responders).

One of the principal objectives of this study was to establish whether the concentration of unusual metabolites excreted was related to the oestrogen-receptor (RE) status of the tumour. Since the data in Table I suggest that similar concentrations of unusual metabolites are excreted by responders (R) and non-responders (NR) to endocrine therapy and, further, since it is known that functional RE is normally associated with responding tumours (Barnes *et al.*, 1979; Leake *et al.*, 1979), a direct correlation between unusual-metabolite excretion and RE concentration is unlikely. Nevertheless, a close relationship between Pattern Index and RE status could be predicted, and appropriate studies are under way.

In conclusion, Pattern Index is a useful index of potential response to endocrine therapy. However, the principal current use for studies of excretion of oestrogen metabolites, as described here, might be in early detection of the disease, especially in high-risk patients, and in monitoring response to therapy, especially in patients presenting with very advanced disease.

We are very pleased to acknowlesge essential financial support from the British Council (R.E.L. and L.C.), the CIBA Foundation (L.C.) and the Italian Association for Cancer Research (M.L.C.). This study was part of a special project supported by C.N.R. Contract No. 79.01546.96 (L.C.). We are very grateful to the various surgeons who have either contributed patients to this study or assessed response to therapy.

### REFERENCES

BARNES, D. M., SKINNER, L. G. & RIBEIRO, G. G. (1979) Triple hormone-receptor assay: A more accurate predictive tool for the treatment of advanced breast cancer? *Br. J. Cancer*, **40**, 682.

BISHOP, H. M., BLAMEY, R. W., ELSTON, C. W., HAYBITTLE, J. L., NICHOLSON, R. I. & GRIFFITHS, K. (1979) Relationship of oestrogen-receptor status of survival in breast cancer. *Lancet*, ii, 283.

BULBROOK, R. D., GREENWOOD, F. C. & HAYWARD, J. L. (1960) Selection of breast cancer patients for adrenalectomy or hypophyesctomy. *Lancet*, i, 1154.

BULBROOK, R. D., HAYWARD, J. L. & SPICER, C. C. (1971) Relationship between urinary androgen and corticoid excretion and subsequent breast cancer. *Lancet*, ii, 395.

CAMPBELL, R. C. (1974) *Statistics for Biologists*, Ch. 6. Cambridge: University Press. p. 135.

CASTAGNETTA, L., TRAINA, A., AGOSTARA, B., D'ALESSANDRO, A. M. & BRUCOLI, G. (1976) Oestrogen excretion patterns in normal post-menopausal women. In *International Symposium on Metastatic Human Breast Cancer*. Ed. Ascoli. Palermo: Cancer Centre Publications. p. 51.

CASTAGNETTA, L. (1979) Oestrogen–androgen balance in human breast and prostate cancer. In *Bladder Tumours and Other Topics in Urological Oncology*. Ed. Pavone-Macaluso *et al.* London: Plenum Press. p. 431.

CASTAGNETTA, L., GRANATA, O., LO CASTO, M. & TRAINA, A. (1980*a*) The ratio between the excretion of oestriol and its stereoisomers as an index of oestrogen metabolism in neoplastic and displastic growth of the human breast. *Ital. J. Biochem.*, **29**, 156.

CASTAGNETTA, L., AGOSTARA, B., DI BENEDETTO, F. and 4 others (1980*b*) Steroid profiles in gynaecomastia vera. Pathophysiology of steroid secretion. *Pan Minerva Med.*, **22**, 93.

DAO, T. (1979) Metabolism of estrogens in breast cancer. *Biochim. Biophys. Acta*, **560**, 397.

EDWARDS, D. P., CHAMNESS, G. C. & MCGUIRE, W. L. (1979) Estrogen and progesterone receptor proteins in breast cancer. *Biochim. Biophys. Acta*, **560**, 457.

FISHMAN, J., BOYAR, R. M. & HELLMAN, L. (1975) Influence of body weight on estradiol metabolism in young women. *J. Clin. Endocrinol. Metab.*, **41**, 989.

HAWKINS, R. A., ROBERTS, M. M. & FORREST, A. P. M. (1980) Oestrogen receptors and breast cancer: Current status. *Br. J. Surg.*, **67**, 153.

KNIGHT, W. A., LIVINGSTONE, R. B., GREGORY, E. J. & MCGUIRE, W. L. (1977) Estrogen receptor as an independent prognostic factor for early recurrence in breast cancer. *Cancer Res.*, **37**, 4669.

KODAMA, M. & KODAMA, T. (1975) Hormonal status of breast cancer. I: Theoretical basis for the analysis of steroid profiles. *J. Natl Cancer Inst.*, **54**, 1023.

LEAKE, R. E., LAING, L. & SMITH, D. C. (1979) A role for nuclear oestrogen receptors in prediction of therapy regime for breast cancer patients. In *Steroid Receptor Assays in Human Breast Tumours: Methodological and Clinical Aspects*. Ed. King. Cardiff: Alpha Omega. p. 73.

LEAKE, R. E., LAING, L., CALMAN, K. C., MACBETH, F. R., CRAWFORD, D. & SMITH, D. C. (1981*a*) Oestrogen receptor status and endocrine therapy of breast cancer: Response rates and status stability. *Br. J. Cancer*, **43**, 59.

LEAKE, R. E., LAING, L., MCARDLE, C. & SMITH, D. C. (1981*b*) Soluble and nuclear oestrogen receptor status in human breast cancer in relation to prognosis. *Br. J. Cancer*, **43**, 67.

LEMON, H. M., WOTIZ, H. H., PARSONS, L. & MOZDEN, P. J. (1966) Reduced oestriol excretion in patients with breast cancer prior to endocrine therapy. *J. Am. Med. Assoc.*, **196**, 1128.

MACMAHON, B., COLE, P., BROWN, J. B. & 4 others (1971) Oestrogen profiles of Asian and North American women. *Lancet*, ii, 900.

MARTUCCI, C. & FISHMAN, J. (1976) Uterine estrogen receptor binding of catecholestrogens and of esterol (1,3,5(10)-estratriene-3, 15$\alpha$, 16$\alpha$, 17$\beta$-tetrol). *Steroids*, **27**, 325.

NIMROD, A. & RYAN, K. J. (1975) Aromatization of androgens by human abdominal and breast fat tissue. *J. Clin. Endocrinol. Metab.*, **40**, 367.

PARAROPOLI, G., CASTAGNETTA, L., TRAINA, A., BRUCOLI, G. & AGOSTARO, B. (1977) Human breast cancer: Studies on urinary excretion of endogenous oestrogens and its changes after hormone treatment. In *Prevention and Detection of Cancer, Vol.* 1, *Part* 1. Ed. Nieburgs. New York: Dekker. p. 627.